# Pharmacists’ Experiences, Perceptions, and Attitudes towards Suicide and Suicide Prevention: A Scoping Review

**DOI:** 10.3390/pharmacy11010025

**Published:** 2023-01-30

**Authors:** Lujain Kamal, Sabrina Anne Jacob

**Affiliations:** Strathclyde Institute of Pharmacy and Biomedical Sciences, University of Strathclyde, 161 Cathedral St, Glasgow G4 0RE, UK

**Keywords:** suicide, pharmacist, attitudes, experience, training

## Abstract

It is important to understand pharmacists’ experiences, stigmas, trainings, and attitudes to suicide, as they can affect the way pharmacists interact with at-risk individuals and influence outcomes. The aim of this scoping review is to explore pharmacists’ willingness, experiences, and attitudes towards suicide prevention, as well as to examine the impact of suicide prevention training programs. A systemic search was conducted using the following databases: PubMed, EMBASE, CINAHL, PsycINFO, Cochrane Central Register of Controlled Trials, and Cochrane Database of Systematic Reviews. Studies included were from database inception to 31 August 2022, in English, with full-text available. A total of 13 studies were included. Training was a key factor which had an impact on pharmacists’ attitudes, experiences, and preparedness to participate in suicide care, with studies revealing the lack of training and the call for more training by pharmacists. Another key factor was closeness to mental illness, which also impacted pharmacists’ attitudes and experiences with at-risk patients. More research is needed worldwide to understand the different barriers and facilitators to pharmacist involvement in suicide care. Targeted training programs should also be developed to not only increase knowledge and competence, but also to address stigma related to suicide.

## 1. Introduction

According to the World Health Organization (WHO), over 700,000 individuals worldwide die by suicide annually. Among 15–25 year olds, it is the fourth leading cause of mortality, and for every suicide completed, there are approximately 20 times more suicide attempts [1] and 135 persons affected by it [2]. In 2019, the cost of suicide deaths and attempts was valued at USD 4.2 trillion in medical, work, quality of life, and value of statistical life costs [3].

The objective of the WHO mental health action plan is to reduce suicide rates by one-third in 2030 [1]. In order to have effective suicide prevention, collaboration among various parts of society is required. A study in the United States (US) reported that about 83% of people who completed suicide received healthcare services the year before their death. However, under 24% received a mental health assessment [4]. Another review found that more than 75% of individuals who committed suicide contacted primary care providers in the year prior to their death, instead of mental health services [5]. This indicates that the primary healthcare sector can play a leading role in suicide prevention. Considering suicide prevalence among patients with mental illnesses, there is an increasing need to train healthcare professionals on mental illnesses and suicide prevention [6].

Published work on healthcare professionals’ experience in suicide have largely focused on physicians and practitioners [6]. One systemic review found that practitioners’ knowledge, attitudes, trainings, and confidence when caring for individuals at risk of suicide can impact outcomes and affect patients’ help-seeking behaviour [7]. Studies have also found that primary health care providers such as doctors and nurses rarely discuss or are reluctant to discuss suicide with patients [8,9]. A study in the Netherlands involving primary care professionals found that barriers to suicide risk assessment included self-incompetence, limited time, heavy workload, and lack of collaboration with mental healthcare [6]. Likewise, a study on nurses’ participation in suicide prevention reported similar barriers to suicide risk assessment and emphasised the importance of training and collaboration among healthcare providers in suicide prevention [10]. A study by Faria et al. found that the training of primary care professionals, which included doctors and nurses, improved participants’ attitudes and interventions when dealing with suicidal patients [11]. Studies that focused on pharmacists’ roles in suicide prevention, however, are lacking. In 2017, a scoping review on community pharmacists and patients at risk of suicide discovered that available literature in this area mainly discussed legal obligations, education, and training [12].

It has been found that close to 90% of people in the US live within 5 miles of a pharmacy [13], 1.6 million people in England visit a pharmacy daily [14], and 350 million people in Australia visit pharmacies annually [15]. Community pharmacies are seen as being very accessible due to their location, extended working hours, and the ability to see a pharmacist and obtain health advice without the need for an appointment [16]. Pharmacists can participate in mental healthcare and suicide prevention ideally by recognising and supporting people experiencing suicidal crises and by restricting access to medications [17], which is the second most common method used in suicide attempts [18]. Indeed, according to the WHO, 20% of suicides are due to self-poisoning [1].

Pharmacists routinely interact with individuals at risk of suicide such as those with mental illnesses, multimorbidity, or going through a crisis; however, little is known about pharmacists’ actual involvement and attitudes towards suicide prevention [12] or individuals’ willingness to reveal their suicidal thoughts to pharmacists. Several studies have suggested that several potential barriers influenced the help-seeking behaviour of suicidal patients such as stigma, fear, embarrassment, and a preference for self-reliance [19,20]. Pharmacy-related barriers were also reported to affect patients’ help-seeking behaviour, for example, workload, lack of time, along with staff negative attitudes and stigma towards mental illnesses [21]. A randomized control trial, however, found that patients with depression accept community pharmacists’ roles in helping them deal with their condition and medications [22]. In the US, Moose and Branham also found that community pharmacists interact with patients with mental health issues an average of 31 times more annually than their primary care providers [23], and patients and carers have reported positive experiences [24]. As for pharmacists attitudes towards providing care for patients with mental illnesses, a cross-sectional survey found that they had positive attitudes when dealing with this population; however, barriers such as lack of confidence and time in managing medications affected their ability to provide optimal care [25].

To increase pharmacists’ involvement in suicide prevention and to improve mental health treatment outcomes, it is crucial to understand and detect the stigma of mental illness and suicide among pharmacists [26]. Additionally, training was identified by other healthcare professionals as an influencer in suicide prevention; however, limited data are available regarding programs provided to pharmacists or their efficacy. Understanding factors that might influence pharmacists’ attitudes when interacting with people at risk of suicide can help increase their engagement in suicide prevention, for instance, their beliefs, cultural background, individual experiences, barriers, or previous training. The aim of this scoping review is to answer two research questions: (1) what are pharmacists’ experiences, perceptions, and attitudes towards suicide and suicide prevention, and (2) what is the impact of training on pharmacists.

## 2. Materials and Methods

This scoping review followed the methodology outlined by Arksey and O’Malley [27]. Reporting for this review was performed in accordance with the Preferred Reporting Items for Systematic Reviews and Meta-Analyses Statement for Scoping Reviews (PRISMA-ScR) [28] (Supplementary Table S1).

### 2.1. Data Sources and Search Strategy

A systemic search was conducted using the following databases: PubMed, Excerpta Medica Database (EMBASE) Cumulative Index to Nursing and Allied Health Literature (CINAHL), PsycINFO, Cochrane Central Register of Controlled Trials, and Cochrane Database of Systematic Reviews. Studies included were from database inception to 31 August 2022. Databases were searched using a combination of MeSH terms and keywords: “pharmacist”, “suicide prevention”, “knowledge”, “experience”, “perception”, “attitude”, and “training”. Search terms were devised by the research team in consultation with the university librarian. Details on the search strategy are provided in Appendix A. A manual search was undertaken to identify additional studies from references cited in retrieved articles. In addition, a free-hand search was undertaken in the following databases: Google, google scholar, BASE, EASY, DuchDuchGo, and arXiv, to obtain a full picture of the search topic. Search terms were “pharmacists and suicide prevention”, “pharmacists’ perception in suicide prevention”, and “gatekeeper and suicide”.

Search results were exported into Endnote 20 (Thomson Reuters, New York, NY, USA) and Covidence, where duplicates were removed. The remaining articles then underwent two levels of screening independently by the authors: (1) title and abstract review and (2) full text review. At both levels, papers were screened to confirm eligibility according to the inclusion and exclusion criteria. Any discrepancies were discussed between both authors.

### 2.2. Study Selection

Studies that met the following inclusion criteria were included: (1) studies reporting pharmacists’ experiences, attitudes, or trainings in suicide prevention and management and (2) studies that involved pharmacists’ interactions with patients who have signs or risk factors for suicide. Studies that met any of the following criteria were excluded: (1) papers reporting only on pharmacy students’ experiences in suicide prevention programs; (2) studies involving multiple healthcare professionals, where pharmacists’ data could not be extracted separately; (3) studies only examining pharmacists’ perspectives in dispensing medications with a high risk of misuse or being used in suicide; (4) studies focusing on pharmacists’ trainings and roles in mental illnesses not related to suicide. Studies not published in English, reviews, systemic reviews, scoping reviews, meta-analyses, in vitro and in vivo studies, animal studies, conference abstracts or proceedings, reports, letters to the editor, comments, and studies where full-text and/or abstract were not available were also excluded.

### 2.3. Data Extraction

The following data were extracted independently by both authors: (1) author, year of publication, country; (2) title and objectives; (3) study design, method, and study instruments used; (4) sample size and characteristics (gender, age, ethnicity, work position, practice location, working hours, practice setting, years in practice); (5) participants’ personal experiences with suicide, training, and mental illnesses; (6) results and outcome. Where full texts were not available, the authors were contacted.

### 2.4. Data Analysis

Data were analysed via thematic narrative analysis. Codes were initially identified, and then, descriptive coding was undertaken to determine categories and themes [29]. Results were presented in tabular format and figures and common themes were identified.

## 3. Results

### 3.1. Study Search

**Figure 1:**
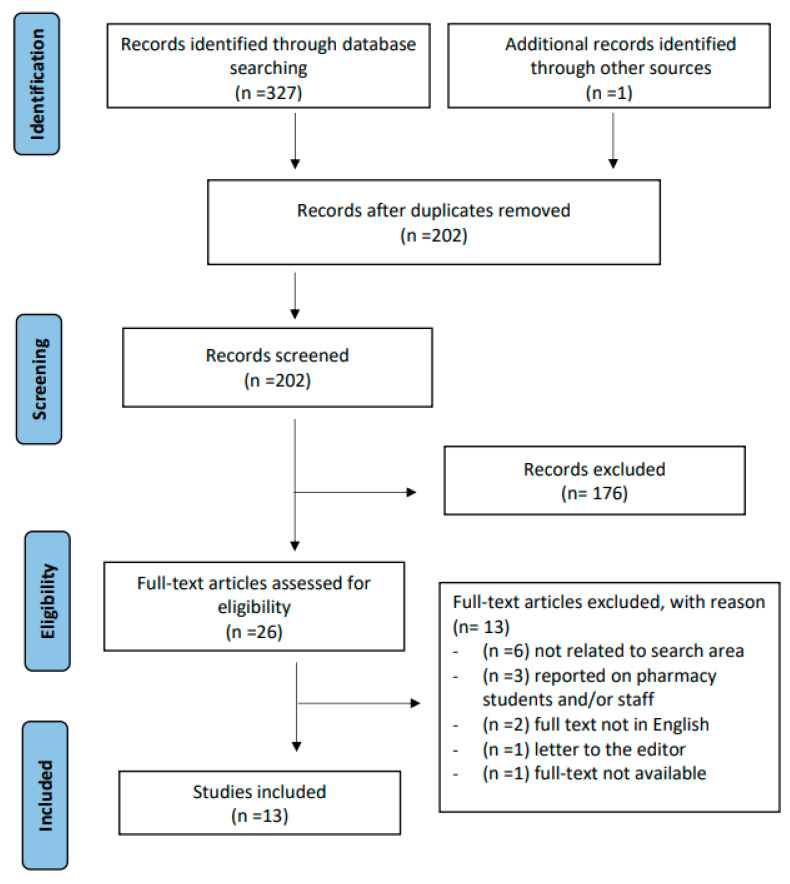
PRISMA-ScR Flow Diagram.

A total of 327 articles were identified from the database search. Following removal of duplicates and screening of titles and abstracts, 26 full texts and their references were screened, and of these, only 13 citations meeting the inclusion criteria were included in this review (Figure 1). The characteristics of included studies are outlined in Table 1.

### 3.2. Study Characteristics

A total of 3039 pharmacists participated in the studies, with close to 70% being females, and the average age was 41 years. Almost 80% of pharmacists worked in the community pharmacy, while approximately 60% practiced in urban areas. Five studies were conducted in Australia and Canada [26,30,31,32,33], five in the United States (US) [34,35,36,37,38], one in the United Kingdom (UK) [39], one in European countries (Germany, Hungary, Ireland, Portugal) [40], and one in Japan [41]. Eleven studies were published from 2017 onward.

Eleven studies employed surveys, with different study instruments utilised. Four studies used the Attitudes Towards Suicide (ATTS) scale [26,30,32,33,37,41], two used the Stigma of Suicide Scale (SOSS) [26,30,32,33], three used the Attitudes to Suicide Prevention (ASP) scale [34,35,38], one used the Pharmacy Suicide Interaction Scale (PSIS) [36], and one study used the Depression Stigma scale (DSS), Intervention Knowledge Test (IKT), and Morris Confidence Scale (MCS) [40]. Four studies employed open-end questions to further explore pharmacists’ experiences or opinions [31,33,36,42]. One study used qualitative interview methods [39].

### 3.3. Key Findings

**Figure 2:**
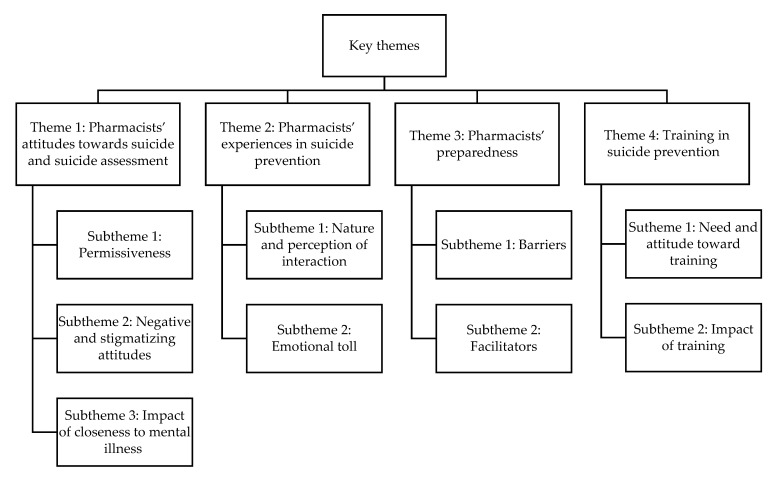
Themes and subthemes.

Four key themes were developed from the included studies: (1) pharmacists’ attitudes towards suicide and suicide assessment, (2) pharmacists’ experiences in suicide prevention, (3) pharmacists’ preparedness and (4) trainings in suicide prevention. These are illustrated below and in Table 1 and Figure 2.

**Table 1:** Summary of included studies.

**Author;** **Year of Publication;** **Country ^a^**	**Objectives ^b^**	**Study Design and Instruments Used**	**Number (n) of Pharmacists;** **Practice Setting;** **Practice Location ^j^**	**Study Population Characteristics (Gender, Age) ^k^**	**Personal Experience ^#^ and Suicide Training**	**Summary of Results**
Kodaka M et al.; 2013; Japan [41]	Explore attitudes of pharmacists participating in BCPP Specialist seminar towards suicide and its association with demographic, occupational, and personal factors	Survey - ATTS ^c^: 37 items	n = 327 59% in psychiatric hospital, 22% in health insurance pharmacies 11% in general hospitals 9% in other settings	67% female M = 43.2 y (SD ± 11.2)	78% had occupational experience and 48% had personal experience with suicide 19% had suicidal ideation 36% received training	68% believed that suicide can be prevented <45% agreed/strongly agreed to being ready to help suicidal people ~60% agreed/strongly agreed suicide is never justified >50% disagreed/strongly disagreed to having a right to complete suicide ~50% “undecided” to whether “*People who talk about suicide do not actually take their lives*” Participants who had previously received suicide training were more likely to have positive attitudes towards suicide prevention (f = 2.73; *p* < 0.05), believe that suicide can be prevented and were ready to help suicidal individuals (*p* < 0.05), believe that suicide is a common occurrence (*p* < 0.05), and to oppose the idea that those who make threats do not actually act on them (*p* < 0.05) Those with a lifetime history of suicidal thoughts had more permissive attitudes towards one’s right to commit suicide (f = 14.76; *p* < 0.001) Gender, age, years in practice, experience with suicidal patients, or knowing someone who died by suicide were not significantly associated with ATTS subscale scores
Coppens E et al.; 2014; Germany, Hungary, Ireland, and Portugal [40]	Improve CFs’ attitudes towards depression, knowledge of suicide, and confidence to detect suicidal behaviour; identify training needs	Single-group pre-and post-test evaluation - DSS ^d^ - IKT ^e^ - MCS ^f^	n = 128	77% female from overall study M = 42 years from overall study		At baseline, pharmacists showed below average knowledge of suicide (M = 3.71 and SE = 0.17) and little confidence to identify suicidal behaviour Pharmacists showed more preserved attitude and benefited most from the training; DSS scores increased from baseline to post-training and follow-up (t(1225) = 2.65, *p* < 0.01) The training program significantly improved the competencies of CF groups including pharmacists across countries, and these improvements were sustained after three to six months
Murphy AL et al.; 2017 Australia and Canada [30]	Determine pharmacists’ attitudes to suicide	Survey - ATTS: 11 items	n = 339 81% in community pharmacy (CP) Urban: 71% Rural: 27% Remote: 1%	73% females M = 43 y (SD ±14)	58% interacted with people at risk of suicide	58% agreed they would consider suicide if suffering from an incurable disease 72% agreed they understand people choosing to die by suicide <30% agreed that suicide is acceptable to terminate an incurable disease >80% disagreed there may be situations where the only reasonable solution is suicide No significant association found between closeness to mental illness (personal experience of mental illness, having a close relationship with someone who completed suicide, or having a close relationship with someone who has a serious mental illness) and the permissive attitude of pharmacists towards suicide
Cates M et al.; 2017; US [34]	Determine if continuing education (CPE) on suicide prevention can positively affect pharmacists’ attitudes towards suicide prevention	Knowledge-based CPE activity with pre- and post-training surveys - ASP ^g^	n = 173 57.7% in CP 34.6% in hospital			The mean total ASP score decreased from M = 33.1 (±SD 4.3) to M = 30.0 (±SD 6.6) (*p* < 0.001) indicating more positive attitudes towards suicide prevention after the educational activity Pharmacists will practice differently after CPE by: - Having more awareness of suicide warnings - Be more willing to assess people at risk of suicide - Be more open to counselling and communicating with people at risk of suicide The study confirmed that pharmacists’ attitudes are sensitive to improvement through focused education
Painter N et al.; 2018; US [35]	Examine the effect of suicide prevention training program on participant’s perception, self-efficacy, and attitude towards suicide prevention	Question, Persuade, and Refer (QPR) Gatekeeper TrainingProgram and pre- and post-training surveys - ASP	n = 103 30% CP 11% outpatient and ambulatory care 8% hospital and inpatient care 2% nursing home 10% academia 21% not in clinical practice 19% other settings	59% female M = 43 y (±SD 16)	45% knew someone who died of suicide 23% received training	100% believed suicide to be preventable Post training: - general perception increased but not significantly - >70% felt confident in identifying signs of suicide or responding appropriately to suicidal patients - >90% confident in ability to listen without judgement - proportion likely to update knowledge increased from 32% to 98% - significant improvement in likelihood of making appropriate interventions (*p* < 0.001) - previous training associated with increased desire to update knowledge of suicide (β = 0.854; t = 2.34; *p* = 0.024) and make appropriate recommendations (*p* = 0.029) indicating a significant association between self-efficacy and training Knowing someone close who had died by suicide (β = –0.487; t = –3.11; *p* = 0.003) and having previous training in suicide prevention (β = –0.640; t = –3.11; *p* = 0.003) associated with less confidence to identify suicidal signs
Murphy AL et al.; 2018; Australia and Canada [31]	Explore the practice experiences of Canadian and Australian community pharmacists’ in caring for people at risk of suicide	Thematic analysis of open-ended comments	n = 176 100% in CP Urban: 70%, Rural: 28%, Remote: 1%	69% female M = 41 y		“Referral and triage” common theme: pharmacists assessed risks, triaged urgency, and made referrals “Accessibility for confiding”: suicidal thinking conveyed to pharmacists via telephone or face-to-face; challenges of phone interactions: (i) unable to fully evaluate patient’s situation, (ii) difficulty balancing own work in pharmacy and focusing on someone in crisis; challenges with face-to-face interactions: (i) comfort of other patients and pharmacy staff when discussing suicide publicly, (ii) managing complex patients in retail environment, (iii) limitations in competency and education in conflict resolution in cases where it escalates; other roles adopted: (i) gatekeeper of medicine supply, (ii) sole discloser regarding suicidality, (iii) support for those affected by suicide, (iv) assessing validity of suicidality “Emotional toll”: (i) frustrated that patient’s needs are not met due to gaps in healthcare system and lack of resources; (ii) disappointment and dissatisfaction with self for not asking directly, some due to fear despite training; (iii) felt ill-equipped to handle these situations due to lack of knowledge and skills, source of dissatisfaction and discomfort; (iv) pharmacists with knowledge, skills, and resources, had positive experiences after helping people with suicide risk, pharmacists with restricted resources (e.g., capacity, time, private areas) were more stressed and dissatisfied; (v) feeling of vulnerability and loss of power due to reliance on others to continue care for patient “Stigma”: felt patients were stigmatized and often labelled as attention-seeking by healthcare professionals and system, resulting in them being turned away without care
Murphy AL et al.; 2019; Australia and Canada [32]	Examine Canadian and Australian community pharmacists’ experiences with people at risk of suicide	Online survey	n = 396 (324 provided details on experience) 87% in CP	70% female	44% interacted with people at risk at least three or more times 18% received training in mental health crisis	66% reported that patients directly indicated having thoughts of suicide; <15% directly inquired about it During assessment, 75% determined the existence of thoughts of suicide, but ~40% or fewer inquired about plans and means to carry them out ~60% felt uncomfortable to very uncomfortable with involvement; 25% felt dissatisfied to very dissatisfied with their management of the situation Barriers to care: lack of training (65%), lack of confidence (43%), not knowing what to do (63%), lack of privacy 58% agreed they were prepared to help a person experiencing a suicidal crisis Belief in preventability positively associated with being prepared to help a person in suicidal crisis (*p* < 0.001) Factors negatively associated with preparedness to help: having a patient who died by suicide (*p* = 0.049), lack of training (*p* = 0.043), lack of confidence (*p* = 0.003), and permissive attitudes towards suicide (*p* < 0.001)
Murphy AL et al.; 2019; Australia and Canada [26]	Measure community pharmacists’ stigma of suicide	Survey - SOSS-SF ^h^: 16 items - ATTS	n = 396 86.6% in CP	70% female M = 38.6 y (±SD 12.7)	72% had a close friend or relative lives with a mental illness 39% had a close friend or relative who attempted suicide or died from suicide 40% interacted with people at risk at least once 30% were ever diagnosed with mental illness 18% received training in mental health crisis	104 who did not know someone close to them living with a mental illness agreed with words describing those who die by suicide as “pathetic” (*p* = 0.008), “stupid” (*p* = 0.007), “irresponsible” (*p* = 0.01), and “cowardly” (*p* = 0.01) 261 who do not have a diagnosis of mental illness agreed with words such as “immoral” (*p* = 001), “irresponsible”(*p* = 0.001), “cowardly”(*p* = 0.02), and “vengeful”(*p* = 0.04) None endorsed the word “immoral” if patients they cared for had died by suicide (*p* = 0.02) With regard to perception of those who completed suicide, most were more likely to agree/strongly agree with the SOSS-SF items for isolation/depression versus stigma and glorification/normalization factors Those with more preventable views of suicide were less likely to endorse stigmatizing terms (*p* = 0.0005) Male gender (*p* = 0.001) and negative perception about suicide preventability (*p* < 0.0005) associated with higher stigma
Gorton H et al.; 2019; UK [39]	Explore current and potential role of community pharmacy teams in self-harm and suicide prevention	One-on-one semi-structured interviews	n = 8	72% female in overall study Majority (55%) >45 years of age in overall study		Overall positive about potential to assist with suicide prevention Many had experience helping those with suicidal thoughts Close relationship with patients facilitated providing help in suicide cases as able to recognise alarm signs and provide immediate support Facilitators: extended opening hours, the lack of need for an appointment, ability to be contacted via phone or in-person and availability of private rooms Barriers: lack of time, resources, and funding for suicide prevention services No training received but welcomed and believed it would help them be more effective as often unsure if what was said was correct; most relied on personal experience Use services such as medication-use reviews to contact and interact with patients at risk Absence of a well-defined referral pathway and two-way communication between pharmacy teams and other healthcare professionals highlighted Importance of restricting access to medications with high risk of poisoning or self-harm, especially if they are supplied against a prescription
Cates M et al.; 2019; US [38]	Determine pharmacists’ attitudes, interests, and perceived skills in suicide prevention	Survey - ASP	n = 227 ~67% in CP ~12.5% in hospital ~20% in other practice settings	63% female 56%: 26–45 y 32% 46–65 y	~20–25% knew someone that died of suicide 33% interacted with people at risk of suicide 4% received training	More disagreement (29%) vs. agreement (25%) in interest in being directly involved in suicide prevention (e.g., screening or counselling at-risk patients) <50% agreed they have an interest in being indirectly involved in suicide prevention (e.g., distributing patient literature) 56% agreed they had an interest in receiving training in suicide prevention The ASP Scale: - 90% reported they lack the necessary training - >50% disagreed to feeling comfortable about asking patients direct, open questions on suicide - Item “*I resent being asked to do more about suicide*” had an M = 1.8 (±SD 0.63), which indicated a positive attitude towards suicide prevention - Items “*It is easy for people not involved in clinical practice to make judgments about suicide prevention*” and “*I don’t feel comfortable assessing someone for suicide risk*” had means of M = 3.28 (±SD 0.9), M = 3.11 (±SD 0.97), which indicated more negative attitudes towards suicide prevention. - Inverse correlation found between ASP scores and participants’ interests in direct involvement in suicide prevention, indirect involvement in suicide prevention, and receiving training in suicide prevention (*p* < 0.001) * - Perceived skills in suicide prevention had statistically significant correlations with interest in being directly involved in suicide prevention (*p* < 0.05)
Carpenter D et al.; 2020; US [36]	Develop and evaluate a measure to assess the frequency that pharmacy staff meet suicide at-risk patients; describe their interactions in suicide prevention and training preferences.	survey - PSIS ^i^	n = 163 100% CP		98% encountered patients at risk 7% had previous suicide prevention training	Only 16% reported counselling about warning labels for the risk of adverse effects of suicidal ideation or behaviour ~25% moderately or extremely uncomfortable talking with at-risk patients Barriers to talking about suicide with patients: fear of making things worse (60%), not knowing what to say (55%), lack of time (50%), fear of offending and liability (~45%), not knowing where to refer patient (43%) 90% expressed interest in attending training on suicide prevention Open-ended comments: training would have helped interaction with at-risk patients, lack of time and reimbursement to have lengthy interaction with patients Experience: no warning before the patient died by suicide, mainly verbal warning signs, e.g., directly expressing intent, asking about lethal dose of medication, efforts made to follow-up with at-risk patients Strategies used with at-risk patients: contacting others, emotional support, keeping them talking until help arrived, listening Some did not know what to do; very few directly enquired about suicide intent
Gillette C et al.; 2020; US [37]	Investigate community pharmacists’ attitudes towards suicide; identify pharmacist-reported barriers to suicidal assessment; evaluate facilitators and barriers to pharmacists conducting suicidal assessments	Online survey - ATTS: 23 items	n = 225 40% chain pharmacy 32% independent pharmacy Urban: 20% Suburban: 40% Rural: 40%	70% female	58% knew someone that died of suicide 67 knew someone who expressed suicidal thoughts 76% knew someone who attempted suicide	95% agreed/strongly agreed mental health conditions and suicidal ideation were real diseases 45% reported that screening for suicidal ideation was a professional responsibility <25% agreed/strongly agreed they knew how to help a patient experiencing suicidal ideation <15% stated they assess for suicidal ideation at least sometimes Most common reported barriers to assessing for suicidal ideation: (i) lack of education in mental health screening, (ii) lack of knowledge, confidence, or skill in performing suicidal ideation assessment, (iii) lack of time ATTS score: - Median 70 (IQR = 7), indicating that pharmacists were undecided about personal attitudes towards suicide - Pharmacists who knew someone who died from suicide had significantly lower median ATTS score than those who did not (69 (IQR = 6) v. 72 (IQR = 7), Z = 3.08, *p* = 0.002)), indicating more understanding or positive attitude towards suicide prevention - Statistically significant association was found between reporting that patients do not want to talk about suicidal ideation with a pharmacist and ATTS score (X^2^ = 15.30, df = 4, *p* = 0.004), indicating negative attitude towards suicide prevention CP more likely to perform a suicidal ideation assessment when they reported a lower number of barriers (OR = 0.70, 99.5% CI = 0.50, 0.97, *p* < 0.005) and when they knew how to help someone who was suicidal (OR = 11.16, 95% CI = 1.75, 71.23, *p* < 0.005)
El-Den S et al.; 2022; Australia and Canada [33]	Explore the impact of providing suicide care on pharmacists and the support needed	Online survey - ATTS - SOSS	n = 378 urban: 73%, rural: 25%, remote: 2%	71% female M = 38.7 y (± SD 12.6)	28% had a patient that died of suicide 84% had interacted with people at risk of suicide 29% were ever diagnosed with mental illness 19% had previous training in mental health crisis management	62% were encouraged by their experience in suicide care to upskill in mental crisis care 72% felt training in mental health crisis management was very important for community pharmacists 54% directly acknowledged/discussed the issue of suicide with patients More likely to report negative effects if previously interacted with patients at risk of suicide (*p* = 0.001) Factors that encouraged upskilling in mental healthcare (all *p* < 0.05): - previous training - previous interaction with patients at risk of suicide - personal diagnosis with mental illness - having a close contact who had attempted or - died by suicide Qualitative analysis on impact: - lack of knowledge to appropriately care for patients - lack of training options despite willingness to be trained - perceived barriers such as time, system, or feeling that suicide care was outside their professional scope - emotional impact and response post-intervention (anxiety, anger, helplessness), as it influenced their practice, left them frustrated, but also drove them to improve referral and follow-up pathway Factors affecting personal help-seeking behaviour: - barriers: self-stigma, professional obligations over personal wellbeing - facilitator: support from colleagues

^a^ US: United States; UK: United Kingdom. ^b^ Board Certified Psychiatric Pharmacy; CFs: community facilitators. ^c^ ATTS: The Attitudes Towards Suicide Scale; measures attitudes about dying by suicide. It has three sections: suicide expressions, attitudes and common beliefs about suicide, and suicidal expressions. Items are rated on a 5-point Likert scale, ranging from 1 (“strongly agree”) to 5 (“strongly disagree”). The total ATTS inventory score is the mean rating for all items. ^d^ DSS: Depression Stigma Scale; it measures stigma associated with depression. It has two subscales: personal and perceived stigma. Responses are measured on a five-point scale (ranging from zero “strongly disagree” to four “strongly agree”), with higher scores indicating higher levels of depression stigma. ^e^ IKT: Intervention Knowledge Test; it assesses knowledge about suicide, using 9 multiple-choice items which measure risk factors and intervention strategies for suicide. The total score is the number of correct responses. ^f^ MCS: Morris Confidence Scale; one item (“I feel confident that I could identify a person at risk for suicide”), that is measured on a ten-point Likert scale ranging from “not at all confident” to “very confident.” ^g^ ASP: the Attitudes to Suicide Prevention Scale; a 14-item scale with 12 negative statements, as well as a positive statement on suicide prevention, and an additional statement about suicides that are considered preventable, which are reverse scored. Scoring is with a 5-point Likert scale from 1 (strongly disagree) to 5 (strongly agree). Total scores range from 14 to 70, with higher scores indicating more negative attitudes. ^h^ SOSS-SF: Stigma of Suicide Scale. Has 16 items (8 stigma, 4 isolation/depression, and 4 glorification/normalization) measured using a 5-point Likert-type scale (*strongly disagree* (1) to *strongly agree (5)*). ^i^ PSIS: Pharmacy Suicide Interaction Scale; 10 items that assess how often pharmacy staff members encountered patients with suicide risk factors or warning signs. Range from 1 (never) to 4 (often), with higher scores indicating more experiences with at-risk patients. ^j^ Urban: belonging to, or relating to, a town or city; rural: far away from large towns or cities; remote: far away from cities and places where most people live, and are difficult to reach ([43]). ^k^ M = : mean; SD: standard deviation. ^#^ Interaction with people at risk or died by suicide and/or personal diagnoses of mental illness. * Higher scores indicate more negative attitudes towards suicide prevention.

Theme 1: Pharmacists’ attitudes towards suicide and suicide assessment

Eight studies discussed pharmacists’ attitudes towards suicide and suicide prevention [26,30,32,35,36,37,38,41]. Some reported more neutral attitudes towards suicide, with pharmacists believing that suicide is preventable [35,41], while some were undecided about their personal attitudes towards suicide [37] and whether people who talk about suicide actually complete it [41]. Most, however, had either positive or negative attitudes towards suicide, as illustrated in subthemes 1 and 2 below.

Subtheme 1: Permissiveness

Five studies discussed pharmacists’ permissive attitudes towards suicide [30,32,36,37,38]. More than 90% of pharmacists agreed that suicidal ideation was a real disease [37], while more than 70% showed understanding for choosing to die by suicide [30]. A survey by Murphy et al. [26] to determine pharmacists’ stigmas of suicide found that when describing those who completed suicide, there was more agreement with the isolation/depression factors (lost, isolated, lonely, disconnected) on the SOSS-SF scale versus glorification/normalisation factors (brave, strong, noble, dedicated). The authors also found that pharmacists who held more preventable views of suicide were significantly less likely to endorse stigmatizing terms [26].

Subtheme 2: Negative and stigmatizing attitudes

Six studies highlighted the negative and stigmatizing attitudes of pharmacists [26,31,36,37,38,41]. There was low agreement with the fact that it is acceptable to complete suicide due to a terminal disease [30], with more than 50% agreeing that suicide is never justified [41] and disagreeing that anyone has a right to complete suicide or that there are situations where suicide would be acceptable [30,41]. There was agreement with using words such as “irresponsible”, “stupid”, “pathetic” and “coward” to describe those who had died from suicide [26]. Male pharmacists (*p* = 0.001) and those who did not believe that suicide can be prevented (*p* = 0.0005) had significantly higher stigma levels [26].

There was also a general expression of discomfort and hesitation at talking to patients at risk or being involved, directly or indirectly, in suicide prevention [32,36,38]. This was complemented by the findings from the online survey by Gillette et al. [37] which employed the use of the ATTS to determine attitudes towards suicide, where less than 50% felt that it was their professional responsibility. It was found that those with more negative attitudes had significantly less interest in being involved in suicide prevention and in receiving training [38].

Subtheme 3: Impact of closeness to mental illness

Five studies highlighted the influence of closeness to mental illness on pharmacists’ attitudes [26,30,37,38,41]. Closeness to mental illness alludes here to the following: interaction or having a close relationship with someone at risk or died by suicide and personal experience or diagnosis of mental illness.

The study by Murphy et al. [30], involving Australians and Canadians, found no significant association between closeness to mental illness and the attitudes of pharmacists; similarly, in the Kodaka et al. [41] study, no significant association was found between the attitudes of Japanese pharmacists and knowing someone who completed suicide. However, the study by Murphy et al. revealed that those who did not know someone close living with mental illness or did not in themselves have a diagnosis of mental illness had significantly more stigmatizing attitudes [26]. Inversely, the influence of knowing someone who died by suicide was found to significantly lower ATTS scores (*p* = 0.002), indicating a positive effect on attitudes towards suicide prevention [37]. Kodaka et al. [41] found that pharmacists with a lifetime history of suicidal thoughts had significantly (*p* < 0.001) more permissive attitudes, while Murphy et al. [26] found that pharmacists would not endorse the word “immoral” if they had lost their patient to suicide.

Theme 2: Pharmacists’ experience in suicide prevention

Six studies discussed pharmacists’ experiences with patients at risk of suicide [31,32,33,36,37,39]. Findings are discussed in terms of the nature and perception of the interaction and the emotional toll on pharmacists due to the experience.

Subtheme 1: Nature and perception of interaction

While many had experience interacting with patients at risk, few enquired directly about suicidal thoughts and plans [32,36,37,41]. Many expressed dissatisfaction with the service provided and were often unsure if what was said was correct [31,32]. Pharmacists in the survey by Carpenter et al. [36] describing their interaction with patients at risk, talked about having no warning before patients completed suicide and shared their experiences with patients asking about lethal doses of medications they were on: “*I had a patient call and ask how much insulin would be a lethal dose. I asked the caller why they were asking that question and the person hung up on me*” [36].

Some of the strategies employed in suicide prevention were providing support, triaging, referring, listening, assessing for ideation, and engaging with patients until help arrived [31,32,36]. It was also suggested that pharmacists use medication review services to interact with patients at risk, other than restricting dispensation of high-risk medications [39]. Having a close relationship with patients was also highlighted as a facilitator, as it enabled pharmacists to recognise alarm signals and provide help immediately [39].

Subtheme 2: Emotional toll

Pharmacists expressed frustration, anxiety, and anger after interacting with patients, mainly with themselves for not knowing what to do or not assessing ideation directly with patients [31,32,33]. This is highlighted in the following feedback: “*I was disappointed in myself as I was afraid to ask her in detail about her plan. I was also hesitant to discuss suicidal ideation with her despite knowing/having done mental health first aid because I didn’t want to worsen the situation/felt very under qualified for this discussion*” [31]. There was also frustration with the healthcare system which was perceived as not meeting the needs of the patient and stigmatised against them [31,33]. In addition, there was a sense of helplessness and vulnerability about having to rely on others to continue the care for the patient [31,33]. Those with more skills and resources reported more positive experiences, while those with limited resources reported more stress [31].

Theme 3: Pharmacists’ preparedness:

Pharmacists’ preparedness to be involved in suicide prevention was mentioned in five studies [32,36,37,38,39,41]. Responses were mixed, with pharmacists in some studies being more prepared and knowing what to do [32,39] compared to others [36,37,41]. Pharmacists with better knowledge on what to do were significantly more likely to assess a patient for suicidal ideation [37], while the lack of training and confidence was significantly negatively associated with preparedness to help [32].

Subtheme 1: Barriers

Two key barriers to pharmacists’ involvement in suicide prevention were identified. The main one was the lack of knowledge, resources, and training in suicide prevention, which resulted in pharmacists not knowing what to say or how to care for these patients [31,32,33,36,37,39]. The second key barrier was workplace related, where pharmacists reported the lack of time and privacy to undertake these conversations and assessments [31,32,33,36,37,39], with one pharmacist lamenting: “…*patient was loud and very upset, I work in a busy pharmacy and it is a difficult setting to address this type of emergency*” [31]. There was also frustration at the lack of a clear referral pathway [17,33,36] with pharmacists underlining the need for the establishment of better referral and follow-up pathways: “*There has to be a better way of follow-up to let these people know someone is looking after them and cares*” [33].

Subtheme 2: Facilitators

Two work environment-related factors facilitated pharmacists’ participation in suicide prevention: the first was the accessibility of pharmacies compared to other general practices such as the lack of need for an appointment, availability of private rooms, and the extended opening hours, which facilitated pharmacists to establish a relationship with patients who visit the pharmacy continuously [39]. This enabled pharmacists to identify alarming suicidal signs and intervene instantly [39]. It also helped some patients to talk to pharmacists regarding their physical or psychological issues [39]. Three studies stated that patients directly talked to pharmacists about suicide, either in person or by phone [31,32,36]. The second factor was pharmacists’ role as gatekeepers of medicine, as they used triage judgment to dispense lower quantities to patients who expressed suicidal signs and used this as a method to keep in contact with them [30]. Pharmacists were also significantly more likely to perform a suicide risk assessment if there were lower numbers of barriers [37].

Theme 4: Training in suicide prevention

Only 20% of pharmacists in the sample received training in suicide prevention. A key factor significantly associated with encouraging pharmacists to upskill in mental health care and crisis was closeness to mental illness (*p* < 0.05) [33]

Subtheme 1: Need and attitude towards training

Pharmacists were mainly positive towards attending training, highlighting its importance in helping them interact with at-risk patients [33,36,38]. This could be attributed to the below-average knowledge on suicide and lack of confidence in identifying suicide behaviour as found in the study by Coppens et al. [40], and the feedback from pharmacists who shared that they were often unsure if what they had said was correct [39]. This sentiment is also captured in the study by El-Den et al. which sought to determine the impact of providing suicide care on pharmacists: “*I realized how unequipped I was to deal with these situations and [it] has helped me want to pursue caring more for these patients and learning more about mental health*” [33].

Subtheme 2: Impact of training

Seven studies found that suicide-related training had positively affected pharmacists’ attitudes towards suicide, including believing that suicide is a common occurrence and rejecting the idea that those who make threats are not serious [33,34,35,38,39,40,41]. Pharmacists reported that training increased their confidence and awareness in recognising suicidal signs, confidence in responding appropriate, confidence in their ability to listen without judgment, and their preparedness and willingness to assess and communicate with those at risk [34,35,41]. It also significantly increased pharmacists’ desires to uptake knowledge in suicide prevention and make proper interventions (*p* = 0.024) [33,35]. In the study by Coppens et al. [40] involving pharmacists from Germany, Hungary, Ireland, and Portugal, training improved pharmacists’ competencies across all countries, and these improvements were sustained after six months. However, one study reported that training was significantly associated with less self-confidence (*p* = 0.003) [35].

## 4. Discussion

Closeness to mental illness, the lack of training and knowledge, and emotional toll affected pharmacists’ experience as well as attitudes. Our findings showed that the majority of pharmacists are not yet prepared to be involved in suicide prevention due to personal barriers such as a lack of confidence and knowledge or system-related barriers such as the lack of time and privacy, workload, and the lack of a clear referral pathway. However, factors that facilitated pharmacists’ participation in suicide prevention included the accessible environment and gatekeeper role. Training improved pharmacists’ attitudes and had a positive influence on pharmacists’ experiences.

From this scoping review, it was found that studies published on pharmacists’ willingness to be part of suicide prevention were lacking, matching the findings of another scoping review in 2017 [12], which suggests the need for further research in this area. There is also a need to assess patients’ opinions about involving pharmacists in suicide prevention. Identified barriers to suicide prevention in this study were similar to those reported in a qualitative study involving primary healthcare professionals where barriers included a lack of time and confidence, heavy workload, and unclear referral pathways [6].

Studies that explored pharmacists’ roles in suicide were mainly conducted in developed countries. No data were found in poor or developing countries, although suicide rates are higher in low- and middle-income countries [1]. This can be a result of mental illness stigma since it is more prevalent in these countries due to cultural or religious beliefs [44]. More research on stigma of suicide and mental illness in developing and poor countries is thus required. Additionally, most published studies focused on pharmacists in community settings, with some extended to other settings, which indicates the need to determine other pharmacists’ attitudes and experiences with suicide, especially those who work in the hospital settings.

Across the included studies, few pharmacists received suicide prevention training, and this was noted to be a key barrier to participating in suicide-prevention activities. Two notable training programmes were offered to pharmacists, the suicide prevention gatekeeper training [31,32,35,38,40,42] and mental health first aid training [31,32,33,37,42]. Even though different training programs were used, this study found that training positively influenced pharmacists’ experiences in suicide prevention. Since suicide prevention training programs are diverse and recent, they need to be evaluated, and training perceived barriers should be assessed. In order to increase pharmacists’ willingness to participate in suicide prevention and to improve their attitudes and experiences in this field, offering a proper training program is suggested. Training programs should address culture barriers and incorporate the following: role of medications in suicide [45], minimizing stigma [26], overcoming barriers, and improving knowledge and communication [37]. The 20 min, Veterans Administration (VA’s) S.A.V.E. training program (signs of suicide, asking about suicide, validating feelings, encouraging help and expediting treatment), might be helpful, as it includes simulated scenarios, with public and phone interactions, that help comprehend skills and attitudes required in such situations [36]. Although the effect of training was sustained over three to six months [40], little is known about the long-term influence on pharmacists’ attitudes, experiences, or their management of at-risk individuals. This matches the findings of another scoping review that explored suicide prevention training programmes given to pharmacists, which noted that additional research is needed to evaluate the impact of training on pharmacists’ self-efficacy and communication skills [45].

This scoping review identified several barriers to pharmacist involvement in suicide prevention, and the first step to increasing pharmacist involvement in this area, other than offering training [46,47], would be to identify and address or offer solutions to these barriers. It has been observed that while pharmacists might feel they should be involved in health promotional activities, the reality of it is very different, and many do not offer such services due to barriers faced [48]. Other methods which can be adopted or ways in which pharmacists can be involved in suicide prevention, as highlighted in this scoping review and other studies, are by assuming the role of a gatekeeper where limited quantities of potentially toxic medication is dispensed to those at risk [30,46], using “referral and triage” systems where a multidisciplinary approach is adopted to refer patients [31,39], using counselling as a tool to develop a therapeutic relationship with patients as well as educate patients on both the pharmacological and non-pharmacological options available for managing mental health issues, provide information to patients on other sources of help for suicidal ideation such as Befrienders, Samaritans, etc., and educating them on the dangers of drug misuse [49], and using medication review sessions to identify those at risk [39,47].

This study had limitations. Three included studies were not solely about pharmacists; thus, demographics and findings could not be extracted. Moreover, studies used different study instruments, and different versions of the same instrument were also used such as the SOSS and ATTS scales. While the end number of included studies was “13”, four of the included papers were reporting on the findings of one study which involved the same participants or respondents [26,30,31,32]. Most studies also involved more than 70% female participants and those below the age of 50, which may have had an impact on responses. In addition, in the study by Coppen et al., only 10% of the participants were pharmacists [40].

## 5. Conclusions

Understanding pharmacists’ attitudes to suicide is important because the way they interact with suicide at-risk patients can affect outcomes and prevent death. Multiple interactable factors can influence pharmacists’ attitudes, either by making it an acceptable choice or by making it a driver to prevent suicide. A proper training program for pharmacists needs to be designed and adapted to increase pharmacists’ preparedness to be involved in suicide prevention.

## Data Availability

The data presented in this study are available on request from the corresponding author.

## Supplementary Materials

The following supporting information can be downloaded at: https://www.mdpi.com/article/10.3390/pharmacy11010025/s1, Table S1: Preferred Reporting Items for Systematic reviews and Meta-Analyses extension for Scoping Reviews (PRISMA-ScR) Checklist. Reference [50] are cited in the supplementary materials.

## Appendix A

**Research strategy:**

▪ Databases used:
  - PubMed;
  - EMBASE;
  - Cochrane Central registration of controlled trials;
  - Cochrane Database of systemic reviews;
  - PsycINFO;
  - CINAHL.
▪ Other sources:
  - Google;
  - Gray literature;
  - BASE;
  - EASY;
  - DuchDuchGo;
  - Arxiv.
▪ Inclusion criteria
  - Studies that reported pharmacists’ experiences, attitudes, or trainings in suicide prevention and management.
  - Studies that involved pharmacists’ interactions with patients who have signs or risk factors for suicide.
▪ Exclusion criteria
  - Studies not published in English;
  - Reviews, systemic reviews, scoping reviews, meta-analyses, in vitro or in vivo studies, studies on animals;
  - Conference abstracts, proceedings, reports, letters to the editor, comments;
  - Studies where full text and/or abstract were not available;
  - Papers reporting only pharmacy students’ experience in suicide prevention programs;
  - Papers reporting on multiple health care professionals, where pharmacists’ data could not be extracted separately;
  - Studies examining pharmacists’ perspectives on dispensing medications with risk of misuse or being used in suicide;
  - Studies focused on pharmacists’ trainings and roles in mental illnesses not related to suicide.
▪ Search terms:
  To obtain the final search terms, we used Mesh PubMed, looked at keywords from related articles, and consulted a librarian and researcher.
  - (People): Pharmacist*;
  - (Outcome): Suicide OR suicide prevention; OR suicide control OR self-killing OR self-harm OR Suicide* Ideation OR Suicide, Attempted; OR completed suicide OR suicide education OR suicide program OR suicide management OR suicide training OR suicidal;
  - (Comparison/behaviour): attitudes OR clinical skills OR knowledge OR experience OR perceptions OR thoughts OR feedback OR opinion* OR sentiment* OR belief OR background OR interaction OR willingness OR confidence OR competent* OR readiness OR behaviour* OR behaviour* OR barrier OR challenge OR benefit OR ability* OR accept*.
▪ Strategy:
  1. Search A;
  2. Search B;
  3. Search C;
  4. Combine A and B and C;
  5. Remove duplicates;
  6. Exclude reviews, scoping reviews;
  7. Screen titles and abstracts for papers about pharmacists’ perception, willingness, and attitude towards suicide prevention.
